# Impulsive and Self-Regulatory Processes in Risky Driving Among Young People: A Dual Process Model

**DOI:** 10.3389/fpsyg.2019.01170

**Published:** 2019-06-12

**Authors:** Lambros Lazuras, Richard Rowe, Damian R. Poulter, Philip A. Powell, Antonia Ypsilanti

**Affiliations:** ^1^Department of Psychology, Sociology and Politics, Sheffield Hallam University, Sheffield, United Kingdom; ^2^Department of Psychology, The University of Sheffield, Sheffield, United Kingdom; ^3^Department of Psychology, Social Work and Counselling, University of Greenwich, London, United Kingdom; ^4^School of Health and Related Research (ScHARR), The University of Sheffield, Sheffield, United Kingdom

**Keywords:** risky driving, young drivers, impulsivity, attitudes, self-regulation, driving violations, driving errors

## Abstract

The present study empirically examined a novel dual process model of self-reported aberrant driving behavior in young and novice drivers that incorporates both impulsive and self-regulatory processes. Four hundred and nine participants aged 18–25 years (*M* age = 21.18 years, *SD* = 2.12; 65.5% females) completed online questionnaires on impulsivity, normlessness, sensation seeking, emotion and self-regulation, and attitudes toward driving safety. Path analysis showed that motor impulsivity was associated with self-reported driving violations, errors, and lapses, whereas sensation seeking was uniquely directly associated with self-reported errors. Non-planning impulsivity, normlessness and sensation seeking had significant indirect effects on self-reported errors, via self-regulation. Finally, motor impulsivity and normlessness had a significant indirect effect on self-reported violations, errors and lapses, via attitudes to driving safety. Based on our findings we suggest that a dual-process approach is relevant to the study of aberrant driving behavior in young and novice drivers, and the results of the present study have important implications for initiatives to promote driving safety in this population.

## Introduction

According to the 2016 World Health Organization report on road safety, over a million people die in road traffic crashes (RTCs) each year, and traffic crash-related injuries represent the leading cause of death among young people aged between 15 and 29 years (World Health Organisation, 2016). In the United Kingdom alone, 29,081 people were killed or seriously injured in 2017 in traffic crashes, with an estimated cost of ∼£16 billion p.a. for reported crashes to the United Kingdom economy (Department for Transport [DFT], 2018). Novice drivers are overrepresented in road traffic casualties, with the per-mile crash rate for teenage drivers being 10 times higher than that of more experienced and older drivers (Mayhew et al., 2003; McKnight and McKnight, 2003). The Decade of Action for Road Safety (DARS) 2011–2020 represents a United Nations initiative that aims to improve road safety and reduce by 50% the number of deaths attributed to traffic injuries and RTCs, especially among young drivers. One of the key action areas of the global plan to achieve the DARS 2011–2020 goals is road user behavior, according to the UN Road Safety Collaboration (World Health Organisation, 2019). Accordingly, a recent evaluation of actual RTC data showed that driver behavior was a main risk factor in 90% of the observed crashes (Dingus et al., 2016). Better understanding the driver-related risk factors for RTCs can help in further promoting road safety in young drivers.

So far, a large body of research has shown that crash involvement has been independently associated with specific types of self-reported aberrant driving behavior, such as intentionally violating traffic rules (e.g., speeding), and unintentional driving errors and lapses (e.g., getting into the wrong lane when entering a roundabout or a junction) while driving (Lajunen et al., 2004; de Winter and Dodou, 2010). Early research suggested that lapses may have consequences for the driver but not for other road users, errors and violations are often hazardous to others, but only self-reported violations were associated with crash involvement (Parker et al., 1995). However, a recent meta-analysis (de Winter et al., 2015) showed that the average correlation coefficient between self-reported violations and crash involvement was 0.13 (based on 67 studies), and the respective correlation with self-reported errors was 0.09 (based on 56 studies), suggesting that both types of self-reported aberrant driving represent risk factors for self-reported RTCs. Previous research has shown that although self-reported violations and errors represent distinct facets of aberrant driving behavior (Lajunen et al., 2004), the correlation between them can be as high as 0.70 (de Winter and Dodou, 2010). Further research into the psychological factors associated with errors and violations is needed in order to identify if similar mechanisms are implicated in the prediction of these two types of self-reported aberrant driving.

### Direct and Indirect Effects of Personality on Risky Driving

One of the most prolific research areas in the psychological study of aberrant driving behavior is concerned with the influence of personality. Different studies have found that emotion-related traits, such as altruism, sensation and excitement-seeking, and hostility were associated with riskier self-reported driving behavior and higher self-reported crash involvement among young and novice drivers (e.g., Ulleberg, 2001; Oltedal and Rundmo, 2006; Machin and Sankey, 2008; Lucidi et al., 2010). Other research has shown that trait impulsivity is particularly relevant to self-reported risky driving in young and/or novice drivers. Impulsivity reflects people’s tendency to act spontaneously and without premeditation and forethought, in response to environmental cues or other triggers, and with a preference for short-term and immediate gratification over long-term and delayed rewards (Moeller et al., 2001). According to Barratt’s three-factor model, trait impulsivity reflects three main characteristics: greater motor activation (motor impulsivity), such as acting at the spur of the moment; less attention to the task at hand (attention impulsivity); and a reduced ability to plan actions (non-planning impulsivity; Patton et al., 1995; Stanford et al., 2009).

Constantinou et al. (2011) used Barratt’s three-dimensional model of impulsivity (Patton et al., 1995; Stanford et al., 2009) to study the associations between motor, attentional, and non-planning dimensions of impulsivity with self-reported risky driving in young drivers. They found that non-planning impulsivity was positively associated with ordinary driving violations. Another study (Hatfield et al., 2017) used both self-reported and lab-based objective measures of trait impulsivity and showed that higher speeding and “riskier” responses (e.g., overtaking, driving through orange lights, not attending to cyclists on the road) in a computerized driving simulation task were positively associated with poorer performance in impulse control tasks (i.e., more errors in a Go/No Go task), but not with self-reported measures of trait impulsivity. Another study found that impulsivity was associated with driving errors (Pearson et al., 2013). Bıçaksız and Özkan (2016) reviewed 38 studies that included measures of trait impulsivity and different self-reported and police-recorded driving outcomes, including driving errors, lapses, violations, driving under the influence, and speeding. They found that, in most studies, aberrant driving outcomes were significantly associated with impulsivity dimensions: “Among the 38 studies reviewed here, impulsivity failed to relate significantly to the driving related measure in any analyses conducted in that study in only four studies” (p. 215).

Previous research has supported the idea that impulsivity and related personality traits are indirectly associated with risky driving outcomes. Sümer (2003) suggested that psychological characteristics, including risk-taking propensity, aggression/hostility and sensation-seeking, create the tendency for aberrant driving behavior (e.g., driving under the influence, speeding, committing errors and violations) which, in turn, can lead to actual crash involvement. The hypotheses derived from Sümer’s (2003) model have been supported by research using self-reported measures of risky driving in young people (e.g., Constantinou et al., 2011). Ulleberg and Rundmo (2003) proposed an alternative model that takes a social cognitive approach and emphasizes the role of safe driving attitudes as a mediator between personality traits and self-reported aberrant driving behavior. According to this model, personality traits related to impulsive behavior and risk-taking, such as sensation seeking, hostility/aggression, and normlessness (i.e., believing that socially unacceptable behaviors are sometimes needed to achieve certain goals), increase the likelihood for aberrant driving (e.g., speeding). On the other hand, traits such as altruism are expected to have a negative correlation with self-reported risky driving and a positive correlation with attitudes toward traffic safety. Ulleberg and Rundmo (2003) further argued that personality traits are likely to have an indirect (vs. a direct) effect on risky driving outcomes, and their model recognizes attitudes to safe driving as a key variable that mediates the association between personality traits and aberrant driving behavior. Their model has been empirically supported by subsequent research studying older (Lucidi et al., 2014) and professional drivers (Mallia et al., 2015). The type of traits associated with self-reported risky driving, and the effect sizes of the direct and indirect associations between personality, attitudes to safe driving, and risky driving indicators (i.e., driving violations, errors, and lapses at wheel) have varied from study to study.

### A Dual-Process Model of Aberrant Driving Behavior

A large body of psychological research has shown that human behavior is not always the product of careful planning, premeditation, and self-regulation of thought and action (Evans, 2008; Evans and Stanovich, 2013; Strack and Deutsch, 2015; Melnikoff and Bargh, 2018b). Rather, automatically activated processes, triggered by apparently mundane environmental cues, can elicit a wide range of unplanned and spontaneous behavioral responses, spanning biased information-processing and social judgments, normative behavior, stereotyping, interpersonal violence and hostility, and risk-taking (Aarts and Dijksterhuis, 2003; Bargh et al., 2012; Rivis and Sheeran, 2013; Sheeran et al., 2013; Melnikoff and Bargh, 2018a). Using the terms coined by Stanovich and West (2000), dual process theorists have categorized automatically activated and consciously controlled higher order cognitive processes into System 1 and System 2 respectively (Evans, 2008). According to this classification, System 1 reflects action driven by impulses, intuition, heuristics, and low mental effort, whereas System 2 is characterized by higher cognitive effort, reflective, and analytic thinking, and is associated with our capacity to control impulses (e.g., inhibitory control), and to regulate our thoughts, emotional arousal, and actions - metaphorically, some scholars have respectively likened System 1 and System 2 processes to “hot” and “cold” reasoning and decision-making (Evans, 2008; Strack and Deutsch, 2015). Although dual process theories were originally developed to account for variations in human reasoning and decision-making processes, the “dualism” concept has found useful applications in other domains, such as understanding health and risk-taking behaviors, and developing relevant interventions to change them (Wills et al., 2011; Hollands et al., 2016; Maher and Conroy, 2016).

The dual process paradigm is highly relevant to understanding (and preventing) aberrant driving behavior in young and novice drivers for the following reasons. Recent developmental neuroscience and neurobiology perspectives posit that neural networks located in the prefrontal cortex (PFC) do not mature until early adulthood, and thus, adolescents and young adults can lack sufficient inhibitory control to resist risk-taking, such as antisocial behavior, substance use/abuse, unsafe sexual activity, and reckless driving (e.g., Kuhn, 2006; Steinberg, 2007; Pharo et al., 2011). This is unsurprising given that the PFC has been described as the “command center” of multiple self-regulatory functions such as inhibitory control, working memory, attentional control and planning, and task switching – collectively known as Executive Functions (EFs) – which are necessary for safe and self-regulated driving (Mäntylä et al., 2009). In addition to neurodevelopmental changes, adolescence and young adulthood are characterized by greater autonomy/independence and spending more time with peers. According to Steinberg (2007, 2010) the still “immature” executive control network cannot sufficiently inhibit impulses and actions driven by the highly active socio-emotional network, which can result in higher risk-taking in the presence of peers. Chein et al. (2011) demonstrated that adolescents, but not adults, committed more errors in a driving-related impulse control (Go/No Go) task when they were tested in the presence of peers, but the amount of errors was not statistically significant between age groups when they were tested alone. Furthermore, in the peer condition, adolescents exhibited greater activation in the brain regions associated with reward valuation (e.g., ventral striatum, orbitofrontal cortex or OFC), and less activation of executive control areas, as compared to adults, and this pattern of activation was significantly associated with greater risk-taking (Chein et al., 2011; Albert et al., 2013).

Other studies using driving simulators have examined the association between EF and driving behavior. One study found that young adults with weaker working memory (updating component) performed worse in a lane changing task during a low-fidelity driving simulation (Mäntylä et al., 2009), and another study found that lower response inhibition was associated with more collisions and slower reaction times to hazards in a medium fidelity driving simulation task (Ross et al., 2015). More recently, Ross et al. (2016) extended their previous research by using a dual process paradigm. They assessed the effects of peer presence on driving performance, and its interaction with executive functions, such as inhibitory control. They found that the presence of peers was associated with greater traffic violations in a driving simulation task - a finding that corroborates previous research on the association between the presence of same-age peer passengers and actual RTCs among young and novice drivers (e.g., Simons-Morton et al., 2011). Ross et al. (2016) further demonstrated that driving violations (e.g., speeding) in the peer presence condition were higher among drivers with lower inhibitory control. To date, the studies by Ross et al. (2015, 2016) are the only ones that have explicitly used a dual system approach to evaluate risky driving in young people using driving simulation tasks. Ross et al. (2016) manipulated peer presence as a primary means to resemble System 1 processes. Peer presence, however, represents the (social) stimuli that may elicit System 1 responses, such as increased risk-taking, and does not necessarily reflect System 1 responses *per se*. Another study showed that neural activity in brain areas involved in response inhibition and cognitive control (i.e., System 2) buffered the effects of peer presence on impulsive risk-taking (System 1) in driving simulation tasks among recently licensed teenage drivers (Cascio et al., 2015). This suggests that System 2 processes play an important role in the way System 1 processes may influence (simulated) driving behavior.

The distinction between System 1 and System 2 may also provide a useful framework on which to model the cognitive bases of driving errors, such as getting into the wrong lane, while driving. Many aspects of car control are likely to rely on the more automatic processes of System 1. However, the avoidance of errors (or lapses) may often require intervention from System 2 at key choice points, in order protect behavior from following the most frequently applied routines in the current situation (Reason, 1990). Impulsivity, and its often identified correlate, inattention, may impinge on System 2 input of this sort.

### The Present Study

Previous research has indicated that a dual process paradigm can be used to better understand the psychological factors associated with RTCs in young and novice drivers (Mäntylä et al., 2009; Ross et al., 2015, 2016). In the present study we propose a dual process model of aberrant driving behavior in young and novice drivers that is differentiated from previous dual process studies of risky driving (i.e., Ross et al., 2016) in terms of methodology and theoretical framework. Specifically, unlike Ross et al. (2016) who utilized a driving simulator, in the present study we assessed aberrant driving behavior with the Driving Behavior Questionnaire (DBQ; Lajunen et al., 2004), a self-reported measure of aberrant driving that has been found to be reliably and validly associated with RTC risk (e.g., near crashes), and self-reported traffic crashes in different cultures and age groups (for a meta-analysis see de Winter and Dodou, 2010; de Winter et al., 2015), and with driver performance in studies using driving simulation tasks (Helman and Reed, 2015). Furthermore, our theoretical proposition is that a dual process paradigm of risky driving in young and novice drivers should take into consideration individual differences that are implicated in System 1 and System 2 processes. These may include impulsivity and related traits (System 1), and self-regulatory capacities (System 2; e.g., emotion and self-regulation).

Our dual-process model is based on three key premises. The first premise is that System 1 (hot) processes are reflected in individual differences in impulsivity, sensation-seeking and normlessness, which have been previously associated with self-reported aberrant driving behavior (Ulleberg and Rundmo, 2003; Constantinou et al., 2011; Bachoo et al., 2013; Berdoulat et al., 2013; Sullman and Stephens, 2013). Accordingly, System 2 (cold) processes are reflected in individual differences in self-regulation (i.e., the capacity to consciously control behavior and restrain impulsive action, Carver and Scheier, 2016), emotion regulation (i.e., the capacity to regulate emotional responses in order to achieve a certain goal), and attitudes toward driving safety, which have been previously associated with lower scores in self-reported aberrant driving (Iversen and Rundmo, 2004; Sani et al., 2017). The second premise of our model is that the distinction between System 1 and System 2 processes respectively resembles the distinction between risk and protective psychological factors for RTCs. This implies that “hot” input from System 1 processes is likely to increase the risk for RTCs, whereas “cold” self-regulatory System 2 processes can mitigate that risk. The third and final premise purports that the effects of impulsivity traits on driving behavior may be mediated by individual differences in self-regulation (see Cascio et al., 2015). To illustrate, young and novice drivers with higher scores in impulsivity and related traits (e.g., sensation-seeking) may respond more emotionally to an environmental cue (e.g., being overtaken) while driving. Whether this emotional response will predict aberrant driving behavior (e.g., driving violations and/or errors) and actual crash involvement will be determined by the driver’s capacity to regulate their thoughts, emotions, and actions and by their attitude toward driving safety - which is hypothesized to be lower in individuals with higher scores in personality traits and characteristics pertaining to System 1, such as impulsivity. With this example the dual-process model of risky driving proposed in the present study explains how impulsivity and impulsiveness-like traits (System 1) are associated with risky driving, and how their effects can be “overtaken” by self-regulatory and reflective processes (System 2). The premises of our model are partly derived from previous theories of self-regulation and impulse control, which purported that self-regulatory failure emerges when the impulsive urges and emotions originating in subcortical structures (i.e., nucleus accumbens/NAcc) cannot be effectively regulated because of diminished self-regulation (i.e., prefrontal activity) due to decision fatigue/ego depletion, negative moods and other influences (e.g., cue exposure; Heatherton and Wagner, 2011).

Based on the aforementioned premises, the following hypotheses were formed.

H1: Trait impulsivity (motor, executive, and planning dimensions), sensation seeking, and normlessness (System 1) will be positively associated with self-reported aberrant driving behavior. Accordingly, self-regulation, emotion regulation and attitudes toward driving safety (System 2) will be negatively associated with aberrant driving behavior.
H2: Emotion, self-regulation, and attitudes to driving safety (System 2) will significantly mediate the association between trait impulsivity dimensions, sensation seeking, normlessness (System 1) and aberrant driving behavior.

## Materials and Methods

### Participants

Overall, 409 students from three Universities in North and South-East England participated in the study. They were aged between 18 and 25 years (*M* age = 21.18, *SD* = 2.12). 65.5% of them identified themselves as females, 87.5% identified themselves as having English/Welsh/Scottish/Norther Irish or British background, their average (median) mileage per week was 20 miles, and the average (mean) time since obtaining their driving license was 7.6 years (*SD* = 2.07).

### Measures

#### System 1 Measures

*Impulsivity* was measured with the Abbreviated Impulsiveness Scale (ABIS; Coutlee et al., 2014). The ABIS is an 11-item measure of trait impulsivity and consists of three subscales that reflect attentional (e.g., “*I don’t pay attention*”), motor (e.g., “*I say things without thinking*”), and non-planning (e.g., “*I am future oriented*” reverse scored item) impulsivity. Responses are coded on a 4-point Likert scale (1 = *rarely/never*, 4 = *almost always/always*). Following reverse scoring of 8 items, a mean score is computed for each subscale and higher scores indicate higher impulsiveness. In the present study, the internal consistency reliability coefficient (Cronbach’s *α*) for each ABIS subscale was acceptable (ABIS non-planning α = 0.71; ABIS motor α = 0.71; ABIS attention α = 0.71).

*Sensation-seeking* was assessed with the mean of five items based on the NEO Personality Inventory (Costa and McCrae, 1992). Responses were recorded on a 5-point Likert scale (1 = *strongly disagree*, 5 = *strongly agree*), and higher scores reflected higher sensation-seeking. Two items (“*I act in a direct way*” and “*I would never go hang gliding or bungee jumping*”) were deleted to improve the internal reliability of the measure, and the final 3-item measure had satisfactory reliability (α = 0.66).

*Normlessness* was measured with the mean score of three items (e.g., “It is ok to get round laws and rules as long as you do not break them directly” and “If something works, it is less important whether it is right or wrong”) based on Kohn and Schooler (1983) normlessness scale, and adapted from Chen (2009) who used this measure in the study of aberrant driving behaviors. Responses were recorded on a 5-point Likert scale (1 = strongly disagree, 5 = strongly agree), and higher scores reflected normlessness. Internal consistency reliability for this measure was satisfactory (α = 0.73).

#### System 2 Measures

*Emotion regulation* was assessed with the Emotion Regulation Questionnaire (ERQ; Gross and John, 2003). The ERQ is a 10-item self-reported survey that measures individual differences in emotion regulation strategies. It comprises two subscales that reflect expressive suppression (e.g., “*I control my emotions by not expressing them*”) and cognitive reappraisal (e.g., “*When I want to feel positive emotion (such as joy or amusement), I change what I’m thinking about*”). Responses are given on 7-point scale (1 = *strongly disagree*, 7 = *strongly agree*), and a mean score is computed for each scale with higher scores reflecting higher emotion regulation in each dimension. The reliability and validity of the ERQ have been reported in previous studies (Gross and John, 2003), and the internal consistency reliability for the ERQ subscales in the present study was high (Cognitive reappraisal α = 0.87; Expressive suppression α = 0.75).

*Self-regulation* was assessed with the 31-item Short Self-Regulation Questionnaire (SSRQ; Carey et al., 2004). The SSRQ reflects different aspects of self-regulation, such as goal-setting and monitoring (e.g., “*I set goals for myself and keep track of my progress*”), deliberate thinking/reasoning of actions (e.g., “*I usually think before I act*”), and self-control (e.g., “*I am able to resist temptation*”). Responses were recorded on a 5-point Likert scale (1 = *strongly disagree*, 7 = *strongly agree*), and a sum score was generated with higher scores indicating greater self-regulatory capacity. The reliability of the SSRQ in the present study was high (α = 0.91).

*Attitudes to driving safety* were assessed with the respective measure developed by Iversen (2004). This is a 16-item self-reported questionnaire of attitudes toward traffic violations (e.g., “*Many traffic rules must be ignored to ensure traffic flow*”), careless driving of others (e.g., “*It’s OK to ride with someone who speeds if others do*”), and driving under the influence (e.g., “*I would never drive after drinking alcohol*”). Responses were recorded on a 5-point Likert scale (1 = *strongly agree*, 5 = *strongly disagree*) and a mean score was computed with higher scores denoting more positive attitudes toward driving safety. The internal consistency reliability of the measure was high (α = 0.81).

#### Driving Behavior Measure

*Aberrant driving behavior* was assessed with the 27-item version of the Manchester Driver Behavior Questionnaire (DBQ; Lajunen et al., 2004), which measured driving violations (11 items, of which 8 items assessed ordinary violations and 3 items assessed aggressive violations), driving errors (e.g., “*On turning left nearly hit a cyclist who has come up on your inside*” - 8 items) and lapses (e.g., “*Misread the signs and exit from a roundabout on the wrong road*” - 8 items). For driving violations, ordinary (e.g., “*disregard the speed limit on a motorway*”) and aggressive violations (e.g., “*Sound your horn to indicate your annoyance to another road user*”) were combined into a single score of “total violations” based on previous research showing that these dimensions could reflect a single factor (e.g., Lajunen et al., 2004), and that aggressive violations do not predict crash involvement independently from ordinary violations (Rowe et al., 2015). Respondents were asked to indicate how often they themselves do each of the violations and errors when driving over the last 12 months or since passing their driving test if that was less than 12 months. Responses were recorded on a six-point scale from “Never” to “Nearly all the time.” Composite scores were computed for each subscale, and higher scores denoted more frequent engagement in each type of aberrant driving behavior. Internal consistency reliability was satisfactory for total violations (α = 0.82), errors (α = 0.76), and lapses (α = 0.72).

### Design/Procedure

A cross-sectional, survey-based design was used to assess the association between System 1 (impulsivity, sensation-seeking, and normlessness), System 2 (emotion regulation, self-regulation, and attitudes to driving safety) and aberrant driving behavior. Participants were contacted either in-person by a trained research associate in University premises, or online through email lists for research participation, and were asked to access and complete an online Qualtrics survey about driving attitudes and behavior. No time restrictions were posed for survey completion and participants took approximately 15 min to complete the online survey. In line with the research ethics guidelines of the British Psychological Society, participants gave their informed consent for participation in the study (by selecting an option in the online survey indicating their agreement to proceed with the study before starting the questionnaire), they were duly informed about the aims and purposes of the study and were given the right to withdraw from it at any point without giving explanations and without any ensuing negative consequences. They were also informed about the anonymity and confidentiality of their responses and were given the opportunity to resolve any questions they had prior to completing the survey. Ethics approval was obtained by the respective ethics review board of the University of Sheffield.

### Data Analysis

Initial inter-correlations (Pearson’s *r*) among study variables were calculated in SPSS v. 24 (IBM Corp., Armonk, NY, United States). Following this a path model was estimated in AMOS v. 24 (IBM Corp., Armonk, NY, United States) to test the study hypotheses, featuring the variables that significantly correlated with the outcome variables (i.e., lapses, errors, and violations). The path model included System 1 traits as exogenous predictors, System 2 traits as potential mediators, and the three outcome variables and modeled the regression parameters simultaneously. Error terms for all endogenous variables were permitted to correlate. Given that the outcome variables were correlated, average effects of each of the predictors on the three outcomes were estimated, as well as the differences in the size of these effects using AMOS custom estimands. Bootstrapping (10,000 resamples; Wood, 2005) was used to estimate the significance of coefficients in the path model (Hayes and Scharkow, 2013). Bootstrapping is incompatible with missing data, so a complete case analysis was conducted (*n* = 307).

## Results

Descriptive statistics and inter-correlations among the study variables are presented in Table 1. The observed correlations were in the expected direction for all measures, thus, supporting the construct validity of the measures used in the study. Taking System 1 measures as an example, sensation seeking was positively correlated with normlessness (*r* = 0.24, *p* < 0.001), attentional (*r* = 0.17, *p* < 0.005), motor (*r* = 0.30, *p* < 0.001), and non-planning (*r* = 0.20, *p* < 0.001) impulsivity. Age was not statistically related to any outcome variable, nor were the emotion regulation variables, and thus these variables were omitted from further analyses.

**Table 1:** Descriptive statistics and inter-correlations among the study variables.

	1	2	3	4	5	6	7	8	9	10	11	12	13	14	15
(1) *Violations*	–	0.55***	0.38***	0.06	–0.14**	0.16***	0.36***	0.17**	0.21***	0.40***	0.15*	–0.06	0.04	–0.16**	–0.56***
(2) Errors		–	0.56***	–0.01	–0.01	0.04	0.31***	0.13*	0.23***	0.21***	–0.04	–0.05	0.06	–0.24***	–0.36***
(3) Lapses			–	0.01	0.11*	0.04	0.27***	0.13*	0.25***	0.12*	0.11*	–0.08	–0.08	–0.20***	–0.21***
(4) Age				–	0.06	0.08	–0.02	–0.02	–0.09	–0.03	–0.14*	0.10	–0.11*	0.11*	–0.03
(5) Sex					–	0.00	–0.11*	–0.14*	0.02	–0.15*	–0.07	0.05	–0.27***	0.07	0.25***
(6) Mileage						–	0.02	–0.06	–0.02	0.15*	0.02	–0.01	0.04	0.05	–0.02
(7) Motor impulsivity							–	0.30***	0.44***	0.21***	0.30***	0.03	–0.02	–0.23***	–0.29***
(8) Planning impulsivity								–	0.50***	0.15*	0.20***	–0.14*	0.00	–0.40***	–0.19***
(9) Attentional impulsivity									–	0.18***	0.17**	–0.23***	–0.05	–0.57***	–0.23***
(10) Normlessness										–	0.24***	0.06	0.22***	–0.25***	–0.50***
(11) Sensation seeking											–	0.16**	0.05	–0.04	–0.16**
(12) Reappraisal												–	–0.00	0.31***	0.06
(13) Suppression													–	–0.15*	–0.19***
(14) Self-regulation														–	0.28***
(15) Attitudes															–

*Mean*	1.85	1.54	2.18	21.18	–	54.24	1.95	1.95	1.99	2.51	3.54	4.69	3.89	108.23	3.69
*SD*	0.59	0.42	0.58	2.12	–	89.57	0.54	0.61	0.50	0.88	0.74	1.12	1.23	16.55	0.56

*^∗∗∗^*p* ≤ 0.001; ^∗∗^*p* ≤ 0.005; ^∗^*p* ≤ 0.05.*

The results of the path model are in Table 2. This model fit the data well, χ^2^(2) = 0.027, *p* = 0.986, CFI = 1.00, RMSEA = 0.00, *p* = 0.994, and this set of predictors explained 17%, 25%, and 42% of variance in the three outcomes, lapses, errors, and violations, respectively. Of the System 1 traits, motor impulsivity was significantly directly associated with all three outcomes with coefficients of a similar magnitude, while sensation seeking was statistically uniquely directly associated with self-reported driving errors. Of the System 2 traits, attitudes was significantly negatively directly associated with all outcome variables, but with a statistically meaningful stronger association with violations than either errors or lapses.

**Table 2:** Path model results for the direct and indirect effects of system 1 traits on self-reported aberrant driving behavior.

	(A) Lapses. *R*^2^ = 0.17, *p* = 0.010			(B) Errors. *R*^2^ = 0.25, *p* = 0.012			(C) Violations. *R*^2^ = 0.42, *p* = 0.006			Average effect			
**Direct effects**	**β**	**BCa 95% CI**		**β**	**BCa 95% CI**		**β**	**BCa 95% CI**		**β**	**BCa 95% CI**		
					
		**LO**	**HI**		**LO**	**HI**		**LO**	**HI**		**LO**	**HI**	**DIFF**

Gender	0.19^∗∗^	0.08	0.30	0.09	–0.03	0.20	0.02	–0.08	0.11	0.10^∗^	0.01	0.18	A > C
Mileage	0.11	–0.04	0.24	0.04	–0.05	0.14	0.17^∗∗^	0.06	0.27	0.11^∗^	0.01	0.20	B < C
(1) Motor impulsivity	0.20^∗∗^	0.06	0.34	0.26^∗∗∗^	0.11	0.41	0.22^∗∗^	0.10	0.35	0.23^∗∗∗^	0.11	0.35	
(2) Attention	0.06	–0.10	0.22	–0.01	–0.17	0.14	0.00	–0.14	0.14	0.02	–0.10	0.14	
(3) Non-planning	0.00	–0.13	0.14	0.00	–0.11	0.11	0.02	–0.09	0.13	0.01	–0.09	0.10	
(4) Normlessness	–0.04	–0.18	0.09	0.02	–0.13	0.16	0.12†	–0.01	0.23	0.03	–0.08	0.14	A < C
(5) Sensation-seeking	0.03	–0.10	0.15	–0.17^∗^	–0.30	–0.04	–0.03	–0.15	0.08	–0.06	–0.17	0.05	A | C < B
(6) Self-regulation	–0.10	–0.24	0.04	–0.13†	–0.26	0.00	0.04	–0.07	0.16	–0.06	–0.16	0.04	B > C
(7) Attitudes	–0.20^∗∗^	–0.32	–0.07	–0.32^∗∗∗^	–0.47	–0.16	–0.46^∗∗∗^	–0.57	–0.34	–0.32^∗∗∗^	–0.43	–0.21	A | B < C

**Indirect effects^a^**													

(1)→(6)	0.00	–0.02	0.01	0.00	–0.02	0.01	0.00	0.00	0.01	0.00	–0.01	0.01	
(2)→(6)	0.05	–0.02	0.14	0.07†	0.00	0.15	–0.02	–0.09	0.04	0.03	–0.02	0.09	B > C
(3)→(6)	0.01†	0.00	0.05	0.02^∗^	0.00	0.05	–0.01	–0.03	0.01	0.01	0.00	0.03	A | B > C
(4)→(6)	0.02	–0.01	0.05	0.02^∗^	0.00	0.05	–0.01	–0.03	0.01	0.01	–0.01	0.03	B > C
(5)→(6)	–0.01	–0.04	0.00	–0.02^∗^	–0.05	0.00	0.01	–0.01	0.03	–0.01	–0.03	0.00	B > C
(1)→(7)	0.03^∗∗^	0.01	0.07	0.05^∗∗^	0.01	0.10	0.07^∗∗^	0.02	0.13	0.05^∗∗^	0.01	0.10	A | B < C
(2)→(7)	0.02†	0.00	0.05	0.03	–0.01	0.07	0.04	–0.01	0.10	0.03	–0.01	0.07	
(3)→(7)	0.01	–0.02	0.03	0.01	–0.03	0.05	0.02	–0.04	0.07	0.01	–0.03	0.05	
(4)→(7)	0.09^∗∗^	0.03	0.15	0.14^∗∗∗^	0.07	0.22	0.20^∗∗∗^	0.14	0.27	0.14^∗∗∗^	0.08	0.20	A | B < C
(5)→(7)	0.00	–0.03	0.02	–0.01	–0.05	0.03	–0.01	–0.06	0.04	–0.01	–0.04	0.03	

**Total effects^a^**													

(1)	0.23^∗∗^	0.09	0.36	0.31^∗∗∗^	0.16	0.44	0.30^∗∗∗^	0.16	0.42	0.28^∗∗∗^	0.16	0.39	
(2)	0.13†	–0.01	0.26	0.09	–0.04	0.21	0.02	–0.11	0.14	0.08	–0.03	0.18	
(3)	0.02	–0.11	0.15	0.03	–0.08	0.14	0.03	–0.09	0.14	0.03	–0.07	0.12	
(4)	0.06	–0.06	0.18	0.17^∗∗^	0.06	0.28	0.31^∗∗∗^	0.19	0.40	0.18^∗∗∗^	0.09	0.26	A < B < C
(5)	0.01	–0.11	0.14	–0.20^∗∗^	–0.32	–0.07	–0.03	–0.16	0.09	–0.07	–0.18	0.03	A | C < B

*N = 307. χ^*2*^(2) = 0.027, *p* = 0.986, CFI = 1.00, RMSEA = 0.00, *p* = 0.994. ^*a*^Analyses control for the effects of gender on the mediators and DVs, and mileage on the DV. BCa 95% CI = Bias-corrected and accelerated bootstrap confidence intervals (10,000 resamples). DIFF = Tests of equivalence between parameters for DV (A), (B), and (C). or = parameters are significantly different at *p* < 0.05. | = parameters are not significantly different at *p* < 0.05. Bootstrapped significance tests are based on standardized coefficients. ^∗∗∗^*p* < 0.001. ^∗∗^*p* < 0.01. ^∗^*p* < 0.05. ^†^*p* < 0.10.*

Of the indirect (mediation) effects of System 1 traits via System 2 traits on driving outcomes, non-planning, normlessness, and sensation-seeking all had a significant indirect effect on reported driving errors via self-regulation, and these effects were statistically stronger than the same indirect effects on reported driving violations. Motor impulsivity and normlessness both had significant indirect effects on all outcomes via attitudes, and these effects were statistically stronger for reported driving violations than errors or lapses.

## Discussion

The present study purported the idea that a dual process paradigm is relevant and useful to the study of the psychological risk factors for RTCs in young and novice drivers - an idea that has started to gain prominence in risky driving research over the last 5 years (see Lambert et al., 2014). We used the System 1/ System 2 distinction (Evans, 2008) to classify impulsivity and impulsiveness traits (i.e., normlessness, and sensation seeking), and self-regulatory capacity (i.e., emotion and self-regulation, and attitudes toward driving safety) respectively, as independent psychological correlates of self-reported driving violations, driving errors and lapses. Accordingly, we hypothesized that self-reported aberrant driving behavior will be positively associated with System 1 and negatively associated with System 2 psychological factors. Our second hypothesis was that the association between System 1 psychological factors (i.e., impulsivity, sensation seeking and normlessness) and self-reported driving behavior (i.e., errors, lapses, and violations) would mediated by self-regulation and attitudes to safe driving, which pertain to System 2 processes. Overall, the results of the study have largely supported our hypotheses in the following ways.

First of all, motor impulsivity was associated with all three indicators of aberrant driving (i.e., self-reported violations, driving errors and lapses). Motor impulsivity reflects behavioral disinhibition (e.g., acting without thinking) and has been associated with impaired inhibitory control (Caswell et al., 2013). Impaired inhibitory control, in turn, has been associated with greater influence of peers on risk-taking in driving simulation tasks among young novice drivers (Ross et al., 2016). Our study corroborates previous findings and indicates that motor impulsivity (behavioral disinhibition) is more relevant to aberrant driving behavior in young and novice drivers, than other dimensions such as executive (non-planning) and cognitive impulsiveness. However, our findings are in contrast with Constantinou et al. (2011) who found that non-planning impulsivity was the only significant correlate of driving violations. A possible explanation is that the association between non-planning impulsivity and self-reported aberrant driving in Constantinou et al. (2011) was attenuated by the inclusion of other predictors, such as sensitivity to punishment/reward and different measures of sensation seeking. Another explanation pertains to the methodological approach used by Constantinou et al. (2011). Specifically, although the zero-order correlations between motor impulsivity and the three dimensions of self-reported aberrant driving were statistically significant (Pearson’s *r* ∼0.17 to 0.27) and comparable to those of non-planning impulsivity (Pearson’s *r* ∼0.09 to 0.22), the authors decided to include only non-planning impulsivity in their path model.

Furthermore, in the present study, the observed effect sizes in the zero-order correlations between sensation seeking and normlessness are in line with those reported in previous research in the context of risky driving in young people (e.g., Lucidi et al., 2010); sensation seeking was associated with driving errors, and normlessness and sensation seeking both had significant indirect effects, via self-regulation, on driving errors. Higher scores in sensation seeking may predispose young drivers to seek excitement in driving, which may, in turn be associated with more driving errors (e.g., braking too quickly on a slippery road) or other risky driving indicators, such as speeding (Machin and Sankey, 2008). Previous research has shown that sensation (or thrill) seeking is associated with both driving violations and driving errors (Jonah, 1997; Wishart et al., 2017). In line with a recent meta-analysis (Zhang et al., 2019), in the present study sensation seeking had a significant but weak correlation with self-reported violations. It is possible that the correlation between sensation-seeking and aberrant driving behavior turns non-significant when a multivariate model is examined that accounts for the effects of other predictors.

We further hypothesized that the effects of impulsivity and related traits (normlessness and sensation seeking) on aberrant driving behavior will be mediated by System 2 self-regulatory processes. The results supported this hypothesis. Specifically, attitudes toward driving safety mediated the associations of motor impulsivity and normlessness with self-reported driving errors, lapses and violations. Accordingly, self-regulation mediated the associations of motor impulsivity, normlessness and sensation seeking with driving errors. Our findings further extend previous research by highlighting the roles of safe driving attitudes and trait self-regulation in mitigating the risk for aberrant driving among young drivers with higher scores in behavioral disinhibition (motor impulsivity). In particular the mediation results would suggest that those with higher System 1 traits are likely to have lower scores in characteristics and traits pertaining to System 2 that would function to regulate their driving behavior.

Self-regulated driving has been mostly studied in the context of older drivers and in association with age-related risk-avoidance and cognitive failure (e.g., Sullivan et al., 2011; Meng and Siren, 2012). To the authors’ knowledge, our study is among the first to report on the effects of self-regulation on self-reported aberrant driving behavior among young and novice drivers. The present findings indicate a protective effect of self-regulation against driving errors, and further showed that the potential risk for traffic crashes that may be posed by impulsive driving can be mitigated by higher scores in driving safety attitudes and self-regulation. It is noteworthy that self-regulation and impulsivity may be related but they represent distinct psychological constructs that have separate functions, neural pathways and developmental trajectories (Hofmann et al., 2012; Nigg, 2017; Steinberg et al., 2018). Furthermore, our findings corroborate past research where better executive functions were associated with reduced risk-taking and risky driving in driving simulation tasks (Mäntylä et al., 2009; Ross et al., 2015). Executive functions play an important role in self-regulatory capacity (Hofmann et al., 2012; Nigg, 2017), and researchers have argued that executive functions allow people to pursue goal-directed behaviors and self-regulate their actions (Dohle et al., 2018). Accordingly, on the basis of the present findings we suggest that self-regulation may be an important explanatory factor in the association between executive functions and driving behavior (e.g., Mäntylä et al., 2009; Ross et al., 2015). It is also possible that the effects of impulsivity on driving behavior are influenced by individual differences in executive functions and self-regulation (Hofmann et al., 2012). However, these hypotheses require further empirical investigation.

The present study is not free of limitations. First of all, we used self-reported measures for impulsivity and aberrant driving behavior. Although self-reported aberrant driving may be subject to socially desirable responses, a large body of research has shown that the DBQ has been significantly associated with self-reported traffic crashes driving behavior in studies using driving simulators (de Winter et al., 2015). Furthermore, significant associations were observed between certain impulsivity dimensions (i.e., motor impulsivity/behavioral disinhibition) and self-reported aberrant driving behavior, and these associations could be examined further with the use of lab-based measures of impulsivity, and more objective measures of risky driving (e.g., valid observations/reports of traffic crashes). Secondly, our study assessed mostly personality traits that reflect System 1 (i.e., impulsive/hot) and System 2 (i.e., controlled/cold) processes. Future studies should incorporate more expansive measures that reflect broader System 1 (e.g., attentional bias to emotional stimuli) and System 2 reasoning and decision-making processes, such as risk perceptions. This will improve our understanding of automatic and reflective traits and processes involved in aberrant driving behavior, and will also lend further support to the idea that a dual process paradigm is needed in order to better understand and more effectively prevent risky driving among young and novice drivers (see Lambert et al., 2014). Also, in our study we used general measures of emotion and self-regulation and the observed effects could be stronger if driving-specific measures were used. Future studies, therefore, may consider the development of driving-specific measures of emotion and self-regulation (e.g., measures that will reflect how well drivers regulate their aggressive thoughts, emotions and action while driving). Finally, we used a cross-sectional design and this poses the inherent problem of reverse causality. Future studies should incorporate a longitudinal design to determine the developmental trajectories of the associations observed in the present study.

With respect to the practical implications of our findings, researchers have recently emphasized the need to reduce health risk-taking by addressing both reflective and automatic processes in reasoning and decision-making (Marteau et al., 2012). We further argue that the time is ripe to consider a dual system approach to risky driving and, accordingly, inform driving safety interventions. Our study showed that the effects of impulsive action can be mitigated by safe driving attitudes and self-regulation. Although future studies are needed to further validate our findings, interventions for driving safety should consider emphasizing the importance of driving safety attitudes, impulse control and self-regulation in mitigating the risks for RTCs among young and novice drivers.

## Ethics Statement

The study reported in the manuscript was approved by the Ethics Review Board of the Department of Psychology, The University of Sheffield with the registration number 130125783, and approval date: 25/11/2016.

## Author Contributions

All authors have contributed equally to the study design and managed the data collection. LL led the writing up of the manuscript. DP, AY, PP, and RR contributed to the editing and revision of the final version of the manuscript. PP performed the statistical analysis.

## Conflict of Interest Statement

The authors declare that the research was conducted in the absence of any commercial or financial relationships that could be construed as a potential conflict of interest.
